# Breast Cancer Among Iraqi Women: Preliminary Findings From a Regional Comparative Breast Cancer Research Project

**DOI:** 10.1200/JGO.2015.003087

**Published:** 2016-03-16

**Authors:** Nada A.S. Alwan

**Affiliations:** **Nada A.S. Alwan**, Baghdad University, Baghdad, Iraq

Breast cancer is the most frequent malignancy among women worldwide, accounting for 25% of all cancers, with an estimated 1.57 million new cases in 2012.^1^ It is also the leading cause of female cancer-related deaths. Although substantial improvement in survival from this disease has been reported in high-resource countries, the risk continues to increase, yielding high mortality rates in middle- and low-income countries.^1,2^ Within the Eastern Mediterranean Region (EMR), cancer is the fourth-ranked cause of death, after cardiovascular diseases, infectious diseases, and injuries.^3^ According to WHO classification, the EMR comprises 21 member states in the Middle East, North Africa, and Central Asia. The included nations are Afghanistan, Bahrain, Djibouti, Egypt, Iran, Iraq, Jordan, Kuwait, Lebanon, Libya, Morocco, Palestinian territory, Oman, Pakistan, Saudi Arabia, Somalia, Sudan, Syria, Tunisia, United Arab Emirates, and Yemen. The International Agency for Research on Cancer (IARC) estimated that 292,677 cases of cancer were newly diagnosed among the female population in EMR during 2012, and 176,139 died of the disease.^1^ The five most commonly recorded cancers in women were those of the breast, colorectum, cervix, ovary, and non-Hodgkin lymphoma. Overall, 99,000 cases were registered as breast cancer in that region.

In addition to being the most important cancer, other features that justify increasing efforts for breast cancer control in the EMR include the exponential rise in the incidence and the higher prevalence of affected young women presenting at advanced stages of disease. These factors led to low 5-year survival rates from breast cancer in many countries within the region as compared with high-income settings.^1,3-9^

Breast cancer has become a major threat to female health in Iraq, where it is the leading cause of death after cardiovascular diseases among women, with a cancer-related mortality rate of 23%.^1,4,8,9^ It has been the highest-ranked malignancy among the Iraqi population in general since 1986. The latest Iraqi Cancer Registry^8^ revealed that among an estimated population size of 32,500,000, a total of 21,101 new cases of cancer were registered in 2012; 9,268 were in men and 11,833 were women.^8^ The crude incidence of all cancers was 61.69 per 100,000 (53.31 in men and 70.59 in women). During that year, 4,115 cases of breast cancer were reported, accounting for 19.5% of all newly diagnosed malignancies and 34% of the registered female cancers, with an incidence approximating 22 per 100,000 female population. The highest frequency was observed in middle-aged women (45-49 years old), whereas the peak age-specific incidence was reported in women 50-54 years old. It has been documented that there is a tendency for the disease to be diagnosed at advanced stages, with a likely prevalence of poorly differentiated tumor forms illustrated in significantly high rates of nuclear aneuoploidy, thus yielding a mortality incidence of approximately 60%.^1,4,8-10^

At the Main Training Center for Early Detection of Breast Cancer in Baghdad, it was reported in 2010 that breast cancer was diagnosed in 19.8% of women presenting with palpable breast lumps.^4^ Although 90.6% of those patients detected the lumps by themselves, only 32% sought medical advice within the first month. Approximately one-third of those patients were diagnosed in their fifth decade of life, 47% presented at advanced stages of disease, and 16% recalled a positive family history.^4^ Another survey that was conducted to explore the level of knowledge, attitude, and practice toward breast cancer and breast self-examination (BSE) among educated Iraqi women demonstrated in 2012 that almost half of the participants had low knowledge scores.^11^ Although 90% of the respondents have heard about BSE, only 43% actually practiced the technique. By multiple logistic regression analysis, researchers found that the level of knowledge among a university-affiliated population in northern Iraq and participants’ age were significantly associated with performing BSE.^12^

The outcomes of those studies obviously illustrate significant knowledge gaps about the relative importance of breast cancer among the Iraqi community and emphasize the urgent need for practical policy decisions to promote early detection through elevating the level of awareness. In general, the poor survival in less-developed countries, including Iraq, is mainly attributed to the lack of strategic, well-designed diagnostic policies coupled with inadequate treatment facilities.^13^

WHO, in collaboration with IARC, organized a consultative Regional Meeting on Cancer Control and Research Priorities in Doha, Qatar, in October 2013. The following recommendations were made: strengthening cancer registration and surveillance, conducting priority research on cancer etiology, and strengthening screening and early detection of priority cancers. It was concluded that the most common cancers amenable to early detection in EMR are those of the breast, colorectum, and cervix, and it was emphasized that strengthening needs to be built on the best international evidence and existing regional experience, taking into consideration the available resources, challenges, and opportunities.^14^

In the context of breast cancer control, information on the putative risk factors for breast cancer and the clinical profile of patients are of utmost importance.^3,15^ Evaluating such processes inevitably demands ensuring the provision of an appropriate sustained database operated by trained personnel. Within hospital records, in the majority of countries belonging to EMR, there is improper documentation of critically important clinical factors such as tumor size, nodal status, stage of breast cancer at initial diagnosis, hormonal receptor status, frequency of distant metastasis, prevailing treatment modalities, and survival. In fact, most of the national cancer registries lack data regarding stage distribution and survival rates.

In an attempt to address these information needs in the clinical profile of patients with breast cancer, and to emphasize the role of research as one of the basic pillars in the adoption of a national cancer control strategy, a national breast cancer research program was established in Iraq in 2009. Under supervision of the IARC Screening Unit, the author developed a comprehensive information system database for Iraqi patients diagnosed with breast cancer. In 2011, the WHO Eastern Mediterranean Regional Office proposed using that model to compare the demographic characteristics, clinicopathologic presentations, and management outcomes among patients in the EMR affected with the disease through implementing a regional comparative breast cancer research project.^10^ An online information system supervised by IARC has been provided to collect data systematically from patients attending targeted breast cancer facilities belonging to eight member nations.

In Iraq, a preliminary analysis of the relevant database findings belonging to 855 patients, diagnosed and treated for breast cancer, documented the following results. Overall, 24.6% of the patients were illiterate, 70.8% sought medical consultation within the same year after detecting abnormal signs or symptoms, and 46% were in their premenopausal age, whereas 35% were diagnosed in the 45-54 year age group. Interestingly, 86.3% were married, merely 7% had their first childbirth after the age of 35 years, and only 8.5% were nullipara. History of lactation and hormonal therapy was reported in 48% and 20.5%, respectively. Overall, 35% documented a positive family history of malignancy, and 18.5% confirmed having relatives with breast cancer. The main presenting signs were palpable breast lumps (94%), skin changes (9.8%), and bloody nipple discharge (4.7%). Bilateral breast cancer was reported in 4.6%. According to TNM classification, 9.8% presented with stage I disease, and 46% were diagnosed in stages III and IV. Infiltrative ductal carcinoma was the most common pathology (67%), followed by intraductal carcinoma (13.6%) and lobular carcinoma (18.5%). Less than 7% of malignant tumors were well differentiated. More than two-thirds of the patients (65.5%) had positive lymph node involvement at the time of initial diagnosis. Immunohistochemical assays demonstrated that estrogen, progesterone and Her2 receptors were positive in 67%, 69%, and 49.2% of specimens, respectively. The majority of patients (92.3%) were provisionally treated by modified radical mastectomy, 35.2% received palliative treatment, hormonal therapy was prescribed to 54.2%, and recurrence was registered in 9.4%.

Comparing our statistics with those reported in high-resource settings (eg, the United States), obvious significant differences are displayed specifically regarding the stage distribution of the disease, with 61%, 32%, and 6% of breast cancer cases present at localized, regional, and distant stages, respectively.^16^ Nevertheless, Iraq, categorized as a middle-income country by WHO/Eastern Mediterranean Regional Office,^17^ documents far better prognostic indicators than those recorded in low-resource settings such as Eritrea in eastern Africa, where two-thirds of the cases are detected in advanced stages; the mean duration from the onset of symptoms to the time of seeking medical advice approaches 3 years.^18^

In general, these findings justify increasing efforts to establish comprehensive breast cancer control programs in Iraq, focusing provisionally on promoting education and early diagnosis as major approaches to controlling the disease. The striking patterns of breast cancer among women in our region highlight the urgent need to consider early detection a priority.
